# Assessing the Accuracy of Property Model Predictions for Cost Optimization of Desalination Technologies

**DOI:** 10.1021/acsestengg.5c00929

**Published:** 2026-01-23

**Authors:** Savannah S. Sakhai, Timothy V. Bartholomew, Alexander V. Dudchenko, Fernando V. Lima

**Affiliations:** † Department of Chemical and Biomedical Engineering, 5631 West Virginia University, Morgantown, West Virginia 26506, United States; ‡ National Energy Technology Laboratory, Pittsburgh, Pennsylvania 15236, United States; § 17220 National Accelerator Laboratory, SLAC, 2575 Sand Hill Road, Menlo Park, California 94025, United States

**Keywords:** desalination, property models, reverse osmosis, mechanical vapor compression, process optimization

## Abstract

Accurate modeling of seawater thermophysical and thermodynamic properties is critical for optimizing desalination processes. This study compares three seawater property models, a Reaktoro multicomponent model, the thermophysical seawater properties library from the Massachusetts Institute of Technology, and a simplified sodium chloride model, in the context of levelized cost of water (LCOW) minimization for reverse osmosis (RO) and mechanical vapor compression systems. Process simulations and cost optimizations reveal that although all three models yield comparable LCOW and specific energy consumption (SEC) estimates under baseline conditions, deviations among their predictions increase with salinity. Relative differences in LCOW and SEC reach up to 6% and 8%, respectively. RO results show greater variability due to differences in osmotic pressure predictions, which affect pressure constraints at high recoveries. Computational performance varies substantially; specifically, Reaktoro simulations are up to 28 times slower than empirical models due to their detailed equilibrium calculations. These results suggest that empirical models offer acceptable accuracy for routine desalination process design, while Reaktoro provides advantages in scenarios requiring detailed speciation, such as scaling or pH adjustment studies. These findings underscore the importance of selecting appropriate property models based on the modeling objective of desalination applications and motivate future work integrating thermodynamic rigor with empirical efficiency.

## Graphical abstract

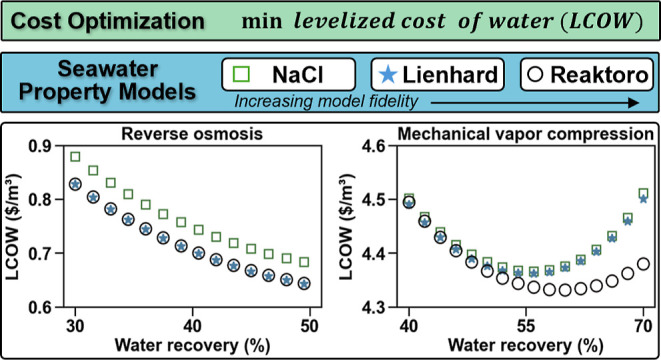

## 1 Introduction

Predicting water properties is essential for process-scale modeling and simulation. In desalination, these properties are vital for understanding the driving forces of separation, such as osmotic pressure in reverse osmosis (RO) and vapor pressure in mechanical vapor compression (MVC). Properties such as specific enthalpy and density are critical for calculating energy balances and solute concentrations. Thus, accurately modeling water properties is crucial for producing dependable analyses of water treatment processes.

A fundamental understanding of these properties has been established for key systems, such as sodium chloride solutions and seawater, which serve as benchmarks for modeling saline water behavior. The properties of sodium chloride solutions are well-studied, including key contributions from empirical and computational studies.^1–4^ Similarly, seawater properties have been extensively documented.^5,6^ Recent efforts have focused on inventorying the literature available regarding the correlations of thermodynamic and thermophysical properties of general saline waters, particularly those dominated by sodium chloride, such as brackish water, seawater, and hypersaline water.^7,8^

While the properties of sodium chloride and seawater are well-characterized, the inherent complexity of water chemistry presents significant challenges that extend beyond these systems. The complex interaction of various dissolved species, chemical reactions, and physical states, all influenced by temperature, pressure, and concentration, necessitates sophisticated property models. Advanced electrolyte models, such as the mixed solvent electrolyte, Pitzer, and electrolyte nonrandom two-liquid frameworks, strive to capture this complexity. While these models are comprehensive, they present significant challenges for these applications. They require solving large, highly parametrized systems of equations and demand extensive input data, which may not always be readily available.

To demonstrate the practical application of such advanced models in real-world systems, recent work has showcased their potential in complex water treatment scenarios. Barber et al. establish a comprehensive flowsheet for a cotreatment process that integrates cooling tower blowdown with produced water, utilizing the OLI Engine to conduct high-fidelity electrolyte thermodynamic calculations.^9^ This methodology enables the accurate modeling of chemical interactions, including solubility predictions and phase equilibria, which are essential for evaluating the complex waters discussed in the work. Ultimately, this facilitates the optimization of the cotreatment process, leading to a reduction in chemical and energy demands while minimizing costs.^10^ Nevertheless, the authors encountered challenges attributable to the complexity of the models, which result in considerable computational time. This example illustrates both the value and the trade-offs of using detailed electrolyte models in process design. While they offer high-fidelity predictions and can significantly improve system optimization, the associated computational burden and the need for specialized tools and expertise often limit their accessibility.

Researchers often resort to simplified property models for analyzing desalination processes. These models bypass detailed water chemistry by tracking salts instead of electrolytes, relying on empirical relationships for property estimation.^11,12^ While computationally efficient, these simplified models are typically tailored to specific water types, such as representative seawater or sodium chloride solutions. This tailoring reduces their applicability to broader water chemistries and limits their accuracy. Mistry and Lienhard specifically investigated the influence of nonideal solution behavior in desalination by evaluating the least work of separation for both ideal and actual sodium chloride solutions, comparing them against seawater.^13^ Their study demonstrated that while the ideal solution approximation introduces errors, these errors tend to cancel out at typical seawater salinities but become significant at very low or very high salinities. They also showed that NaCl solutions more closely approximate seawater behavior as salinity increases. Their findings highlight the limitations of simplified property models in capturing nonideal solution behavior, reinforcing the need for accurate property models in desalination modeling. While their work establishes a strong theoretical foundation for understanding nonidealities in desalination, its direct implications for practical process modeling and cost optimization remain less explored. Their study primarily focuses on thermodynamic limits, whereas real-world desalination systems must balance accuracy with computational feasibility.

Expanding on these insights, this study examines how property model selection influences desalination system design and economics by isolating the effect of property model fidelity on optimization outcomes. By evaluating how these prediction differences propagate through the optimization framework, the analysis aims to bridge the gap between computational efficiency and predictive accuracy for more reliable and scalable process optimization.

## 2 Methods

Cost optimization models for RO and mechanical vapor compression MVC were implemented to encompass evaporative and membrane desalination technologies. In the RO system, illustrated in Figure 1A, a pump feeds into a single-stage RO unit. The MVC system, shown in Figure 1B, utilizes a feed pump to direct the feed through the distillate and brine heat exchangers to the evaporator. The feed is sprayed onto the heat exchanger tubes in the evaporator, causing a portion to vaporize while leaving a concentrated brine behind. The pure water vapor is then compressed and routed through the evaporator tubes to supply heat for the vaporization of the feed. Heat recovery occurs from the distillate and brine in their respective heat exchangers to preheat the feed.

**Figure 1:**
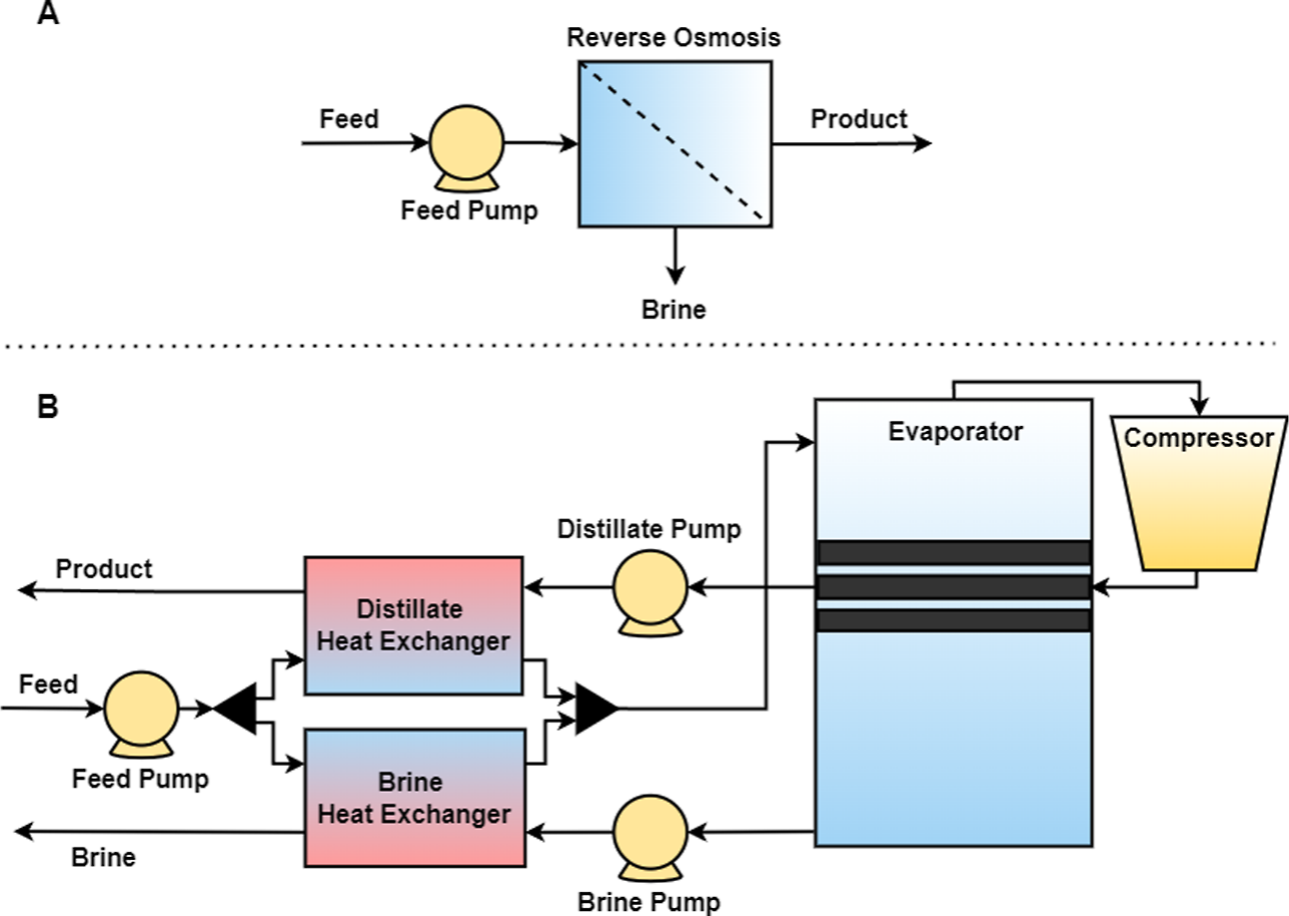
RO and MVC process diagram.

### 2.1 WaterTAP

The cost optimization models are implemented in the Water Treatment Technoeconomic Assessment Platform (WaterTAP) software platform. WaterTAP is a techno-economic assessment platform that simulates, optimizes, and analyzes water treatment trains.^14^ It is a modular model library encompassing unit, property, and costing models. Built on the Institute for Design of Advanced Energy Systems (IDAES) Integrated Platform and Pyomo software packages, this open-source Python package employs equation-oriented solution strategies and interfaces with diverse open-source and commercial optimization solvers, delivering robust computational capabilities for optimization.^15–17^ This work utilizes the WaterTAP unit, property, and cost models to construct each case for analysis. The following sections outline the modeling details in the specified order.

### 2.2 Unit Models

#### 2.2.1 Reverse Osmosis

The reverse osmosis unit model utilizes a one-dimensional approach based on the solution-diffusion model and film theory. Hydraulic and osmotic pressures, along with the concentration gradient across the membrane, are the primary drivers of water and salt fluxes. The governing mass and momentum balances apply a finite difference approximation. The model considers concentration polarization and pressure drop. A single stage appears with continuous length and width variables that characterize a system with discrete spiral-wound modules. The model formulation details are provided in refs 18–20 . The pump unit model calculates power consumption based on flow rate, pressure change, and efficiency.

#### 2.2.2 Mechanical Vapor Compression

The MVC system includes the evaporator, compressor, and complete condenser unit models. The evaporator unit model maintains mass and energy balances. The complete condenser connects to the evaporator through a heat transfer balance, wherein heat transfer occurs between the condensing distillate in the tubes and the vaporizing feed passing over the tubes in the evaporator. The compressor is based on isentropic efficiency. The details of the MVC model formulation are provided in ref 12. The key modeling assumptions include the following: the vapor is pure water, heat losses are negligible, and heat transfer coefficients remain constant regardless of material.

The heat exchanger unit model governs the heat transfer balance between the shell and tube based on the temperature difference, heat transfer coefficient, and heat transfer area. The pump units are modeled as mentioned in Section 2.2.1.

### 2.3 Property Models

#### 2.3.1 Reaktoro Seawater Property Model

Reaktoro is an open-source software developed to provide a computational framework for simulating chemical reactions, equilibrium, and kinetics.^21^ This allows for estimating a given system’s aqueous and thermodynamic properties. Reaktoro performs calculations using PhreeqC, Supcrt, ThermoFun, and National Aeronautics and Space Administration thermodynamic databases and activity models to represent nonideal behavior accurately. The validity range of the calculations is specific to the database and activity model used. For this work, a combination of the PhreeqC (1–1000 bar and 0–200 °C) and Supcrt (1–5000 bar and 0–1000 °C) databases was employed, along with the Pitzer activity model.^22,23^ This decision was based on data availability, as certain key properties were not available in a single database. Specifically, the PhreeqC database was used for the RO case to obtain osmotic pressure and density, while the Supcrt database was used for the MVC case to obtain vapor pressure and specific heat capacity for specific enthalpy calculations. The defined feed composition can be found in Table 1, which shows the major ion composition of typical seawater.^13^

**Table 1:** Major Ion Composition of Typical Seawater^13^

**component**		
**name**	**symbol**	**concentration** **(mg/L)**
chloride	Cl^–^	18,980
sodium	Na^+^	10,556
sulfate	SO_4_^2–^	2649
magnesium	Mg^2+^	1262
calcium	Ca^2+^	400
potassium	K^+^	380
bicarbonate	HCO_3_^–^	140
total dissolved solids		34,367

For the integration of Reaktoro in WaterTAP, an additional package, Reaktoro-pse, was used.^24^ Reaktoro-pse enables the configuration of Reaktoro equilibrium problems as a gray-box model within WaterTAP. This allows for a seamless connection, automating data transfer between Reaktoro and WaterTAP variables.

This property model serves as the basis for comparison within this analysis due to the complexity inherent in its calculations and its ability to account for detailed chemical speciation and nonideal solution behavior. However, it is important to clarify that Reaktoro, while utilized as the comparative benchmark in this study, is not regarded as a universal ground-truth model. The comparisons and conclusions drawn in this work are therefore most directly applicable to seawater-like matrices within the investigated temperature (25–95 °C), pressure (1–85 bar), and salinity ranges (5–125 g/kg).

#### 2.3.2 Thermophysical Properties of Seawater

The seawater property model implemented in WaterTAP was originally developed by the Lienhard Research Group at the Massachusetts Institute of Technology, hereafter referred to as the Lienhard model.^5,6^ The proposed empirical relationships are based on experimental data derived from synthetic and natural seawater, with valid temperature, pressure, and salinity ranges of 0–120 °C, 0–120 bar, and 0–120 g/kg, respectively. The fundamental assumption underlying this model is that a bulk measure of total dissolved solids can effectively capture the characteristics of natural seawater.

#### 2.3.3 Sodium Chloride Property Model

The sodium chloride property model is an empirical model derived from a synthesis of established public data sources.^1,3,25^ The model equations are polynomial and dependent on temperature and concentration, following the structure proposed in ref 3. The influence of pressure on the thermodynamic properties is minimal, particularly in desalination applications, and may thus be neglected.^5^ The valid ranges for temperature and salinity are 0–150 °C and 0–150 g/kg, respectively. This model assumes a sodium chloride solution as a representation of seawater, given that it constitutes the most prevalent component.

### 2.4 Cost Models

The cost models connect capital and operating costs with the system’s design and function. Capital costs depend on the size of the equipment and the materials of construction. The overall investment cost is calculated by adding all direct equipment expenses and multiplying by an investment factor to cover indirect capital costs. Operating costs consider annual electricity consumption, maintenance, and labor expenses. All costs are given in 2020 U.S. dollars.

### 2.5 Case Study Parameters

The cost-optimal design and operation are found for treating seawater across a range of feed concentrations and water recoveries specific to each unit’s application space. For RO, feed salinities of 5–0 g/kg span brackish to high salinity seawater, with recoveries of 35–65% constrained by membrane pressure and fouling. For MVC, salinities of 35–125 g/kg cover seawater through hypersaline brines representative of MLD/ZLD applications, with recoveries of 45–75% limited by brine solubility and scaling potential. These ranges enable evaluation of property model performance across the full spectrum of desalination salinities, from dilute to concentrated conditions. For each unit, three cases corresponding to each property model are considered. All cases use the same process and financial parameters, as detailed in Table 2.

**Table 2:** Process and Financial Parameters Used in the Cost-Optimization Models for the RO and MVC Case Studies

**process specifications for RO**	**value**	**unit**	**source**
**pump**			
efficiency	0.8	-	-
**RO**			
membrane water permeability coefficient	4.20 × 10^–12^	m/s-Pa	^26^
membrane salt permeability coefficient	3.50 × 10^–8^	m/s	^26^
channel height	2.00 × 10^–3^	m	^18,19^
spacer porosity	0.75	-	^18,19^
permeate pressure	1.01 × 10^5^	Pa	-
maximum pressure	8.50 × 10^6^	Pa	-

**process specifications for MVC**	**value**	**unit**	**source**
**pumps/compressor**			
efficiency	0.8	-	-
**preheater**			
heat transfer coefficient	2000	W/K-m^2^	^12^
pressure drop	7000	Pa	^12^
**evaporator**			
heat transfer coefficient	3000	W/K-m^2^	^12^
temperature	75	°C	^12^

**economic parameters**	**value**	**unit**	**source**
electricity cost	0.07	$/kWh	
utilization factor	0.9	-	^18,19^
material factor cost	5	-	^18,19^
factor membrane replacement	0.2	fraction of membrane replaced/year	^18,19^
membrane cost	30	$/m^2^	^18,19^
factor total investment	2	investment cost/equipment cost	^18,19^
factor maintenance labor chemical	0.03	fraction of investment cost/year	^18,19^
factor capital annualization	0.1	fraction of investment cost/year	^18,19^

The comparison focuses on how each property model’s predictions influence the cost-optimal process design, holding all other modeling assumptions constant across cases. This approach isolates property-model effects from other sources of uncertainty in process simulation.

## 3 Results and Discussion

### 3.1 Differences in Property Model Predictions for Key Properties

Key properties for each technology were selected for detailed analysis. For RO, osmotic pressure is a critical property, as it directly impacts the driving force for water transport across the membrane. For MVC, enthalpy and vapor pressure are crucial in determining energy balances. The predictions of each property model were compared over a temperature range of 25–95 °C and salinity levels of 35–150 g/kg.

Figure 2A illustrates the osmotic pressure predictions of the Reaktoro seawater model, showing an increasing trend with both temperature and salinity. Comparing these results with the Lienhard seawater predictions (Figure 2B) reveals relatively minor differences, a 0–4% increase in osmotic pressure predictions. The largest deviations occur at high salinity levels; specifically, when the salinity exceeds the Lienhard model’s upper validity limit of 120 g/kg. The sodium chloride model (Figure 2C) shows larger deviations, approximately double that of the Lienhard seawater model, particularly at low salinity and elevated temperatures. This behavior is attributed to the presence of divalent ions in seawater, which lowers the osmotic pressure below that of a pure sodium chloride solution (see also Figure 5B).

**Figure 2:**
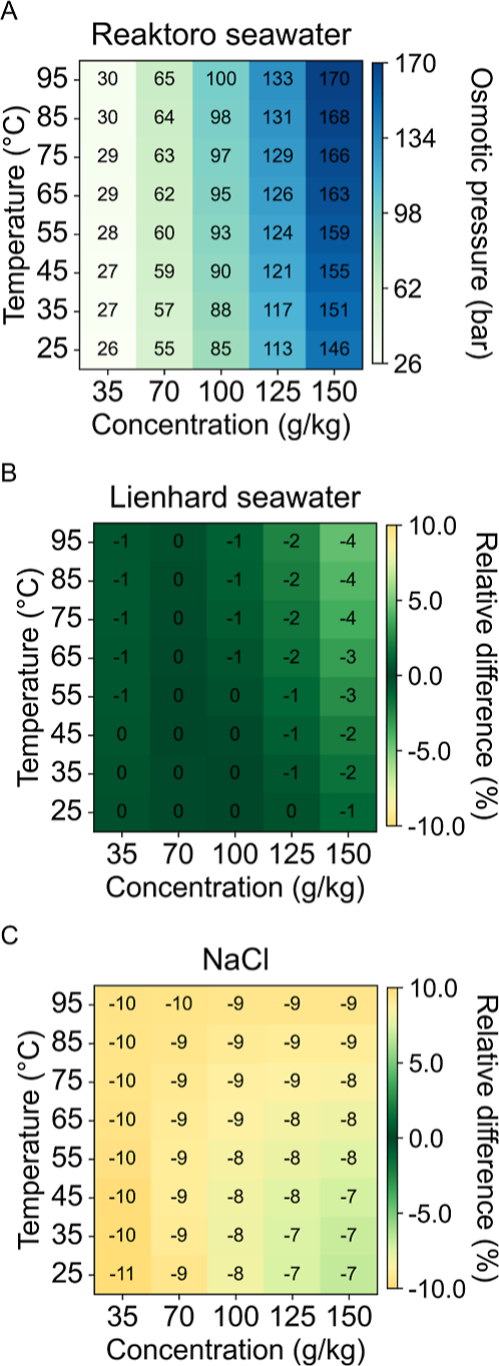
Osmotic pressure predictions for (A) Reaktoro seawater property model and the relative difference of (B) Lienhard seawater property model and (C) sodium chloride property model when compared to Reaktoro.

As shown in Figure 3A, specific enthalpy predictions with Reaktoro increase with temperature and decrease with concentration. The Lienhard seawater model (Figure 3B) exhibits larger differences relative to Reaktoro, a 1–12% decrease in specific enthalpy predictions. The differences are more pronounced at higher temperatures and salinities, indicating limitations in the empirical correlations used. In contrast, the sodium chloride model (Figure 3C) shows more minor deviations, generally within a 3% increase in specific enthalpy predictions, indicating that seawater and sodium chloride solutions at the same salinity yield similar specific enthalpy values.

**Figure 3:**
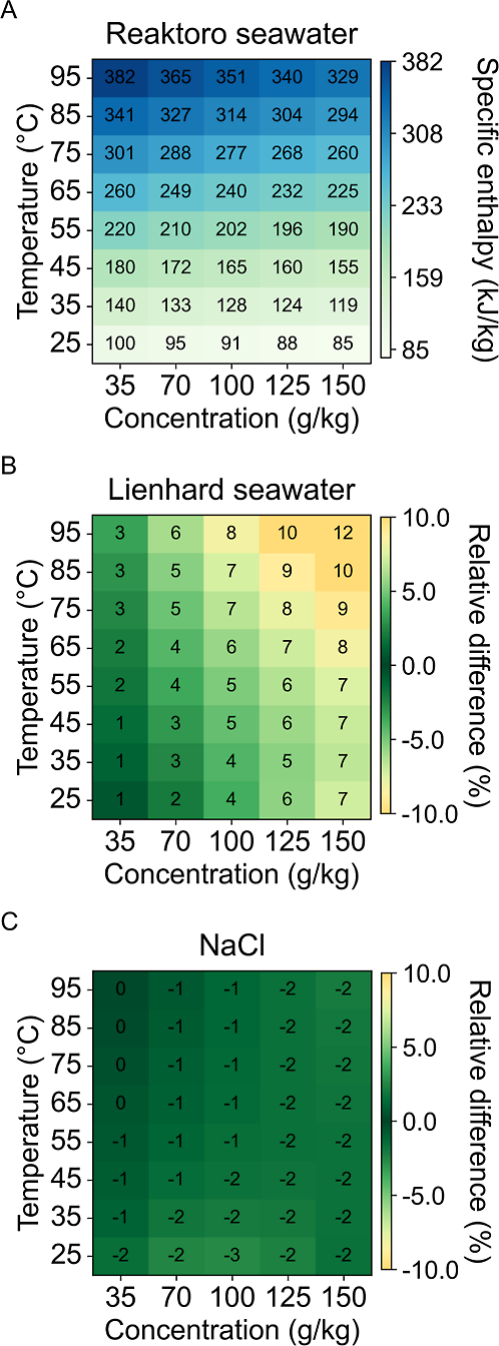
Specific enthalpy predictions for (A) Reaktoro seawater property model and the relative difference of (B) Lienhard seawater property model and (C) sodium chloride property model when compared to Reaktoro.

In Figure 4A, the predictions of vapor pressure utilizing Reaktoro demonstrate an increase with temperature and a decrease with concentration. The Lienhard seawater model, as illustrated in Figure 4B, exhibits negligible differences when compared to Reaktoro, a 0–2% decrease in vapor pressure predictions. The sodium chloride model, as depicted in Figure 4C, demonstrates differences within a 6% decrease in vapor pressure predictions. The differences between the sodium chloride and seawater models can be attributed to nonideal ion interactions. While vapor pressure is typically regarded as a colligative property, at elevated concentrations, the specific types of ions begin to influence the effective concentration.

**Figure 4:**
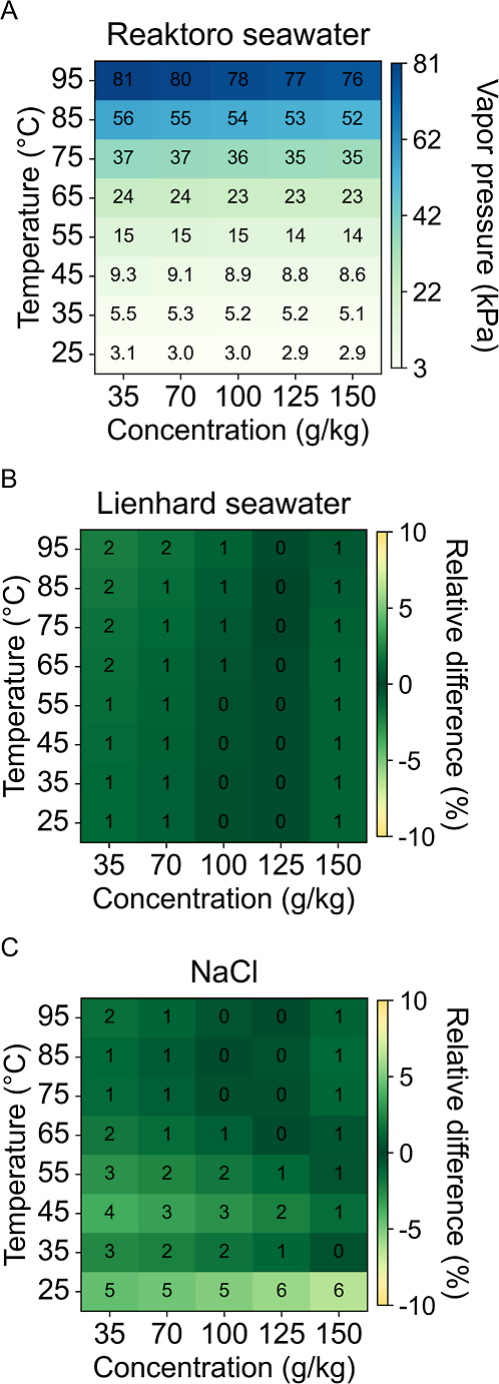
Vapor pressure predictions for (A) Reaktoro seawater property model and the relative difference of (B) Lienhard seawater property model and (C) sodium chloride property model when compared to Reaktoro.

The impact of ionic composition on water properties was further investigated by comparing seawater, monovalent-dominant water, and divalent-dominant water using Reaktoro. Solutions containing sodium chloride and calcium chloride were formulated with divalent-to-monovalent ion ratios of 2:1 and 1:2 to generate representative compositions. Figure 5 illustrates the variation in osmotic pressure and enthalpy with salinity.

**Figure 5:**
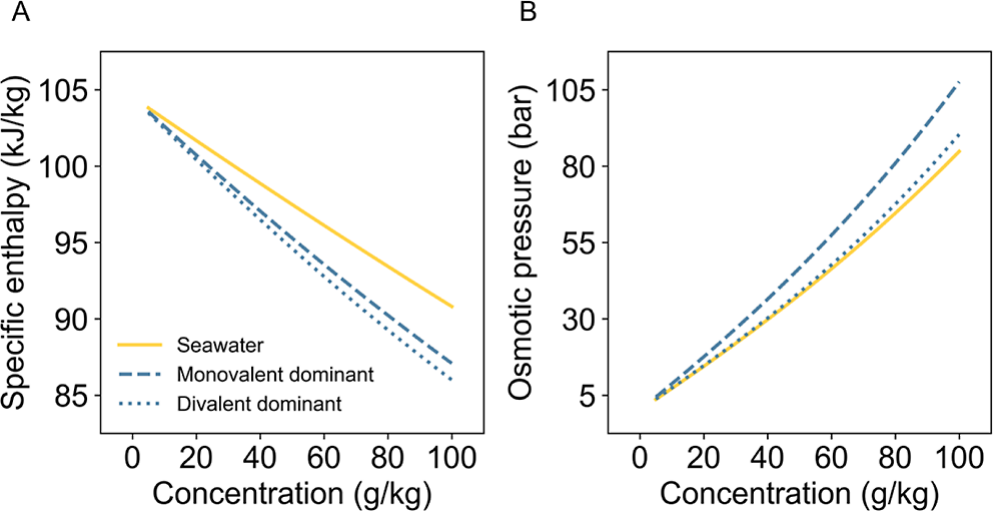
Comparison between seawater, monovalent dominant water, and divalent dominant water for both (A) enthalpy and (B) osmotic pressure calculated via Reaktoro.

As shown in Figure 5B, monovalent-dominant water exhibits higher osmotic pressure than seawater, whereas divalent-dominant water approximates seawater behavior. For divalent-to-monovalent ratios exceeding 2, osmotic pressure decreases below seawater values. Figure 5A shows specific enthalpy trends, where both monovalent- and divalent-dominant waters deviate from seawater. As concentration increases, enthalpy decreases, but each water type declines at a different rate.

These trends in Figure 5 arise from solute–solute and solute–solvent interactions that drive nonideal behavior. Divalent ions have higher lattice energies, greater ionic strength, and stronger ion–ion and ion–water interactions than monovalent ions. This leads to significant ion pairing and reduced free ion availability in solution, decreasing the effective concentration. This results in lower osmotic pressures and a steeper decrease in enthalpy as concentration increases. In contrast, monovalent ions have lower lattice energies, less ionic strength, and weaker ion–ion and ion–water interactions than divalent ions. This results in a solution primarily composed of free ions. Consequently, monovalent-dominant waters exhibit higher osmotic pressures and a more gradual enthalpy decrease with concentration.

### 3.2 Difference in Outcome Metrics and Decision Variables for Process-Scale Cost Optimization

#### 3.2.1 Reverse Osmosis Cost Optimization Results

The model minimizes the levelized cost of water (LCOW) by optimizing the key decision variables. For RO, key variables include the operating pressure, which dictates energy requirements and overall process performance, and the membrane area, which governs system capacity. Considering these decision variables, the evaluation aims to quantify specific outcome metrics that assess the performance and the process design. The performance metrics include the LCOW and the specific energy consumption (SEC), which provide insights into the energy efficiency of the desalination system. Lower SEC values indicate a more energy-efficient process, translating to reduced operating costs. The selected process metrics focus on the design and operating variables; specifically, decision variables, i.e., the membrane area and the operating pressure. An understanding of these metrics allows for a more nuanced analysis of how changes in the property model affect the resulting optimal design.

The results from the RO case study are illustrated in Figure 6, which presents the LCOW outcomes for three distinct seawater property models: (A) the Reaktoro seawater model, (B) the Lienhard seawater model, and (C) the sodium chloride model. Overall, the cost optimization outcomes across the application spectrum show a maximum 1% decrease in LCOW for the Lienhard seawater model and a maximum 6% increase in LCOW for the sodium chloride model compared to Reaktoro-based calculations. The relative differences tend to increase with higher concentration levels, likely due to the mirrored differences observed in osmotic pressure. Given the variations in osmotic pressure predictions among the models and the RO maximum operating pressure limit of 85 bar, the sodium chloride scenario of 45% recovery at a 50 g/kg feed concentration exceeds this limit, which explains why no result is shown for this case.

**Figure 6:**
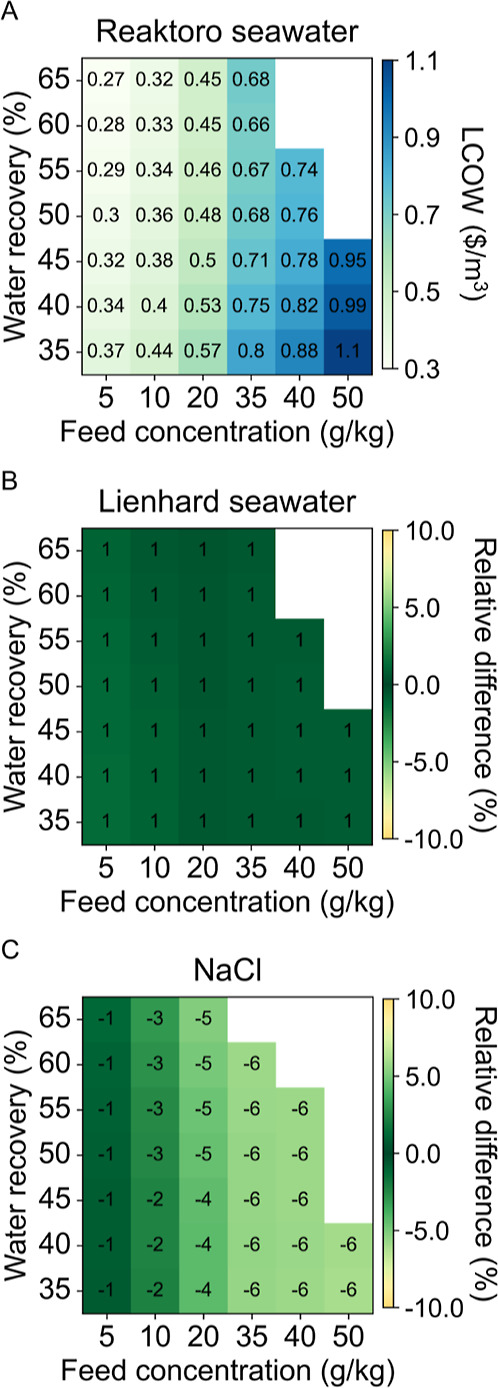
LCOW of RO case study for (A) Reaktoro seawater property model and the relative difference of (B) Lienhard seawater property model and (C) sodium chloride property model when compared to Reaktoro.

The SEC of the RO case study is shown in Figure 7 for each of the three property models considered. The deviations among the property models for the SEC are similar to the predictions for LCOW, with a maximum 2% decrease in SEC for the Lienhard seawater model and a maximum 7% decrease for the sodium chloride model when compared to Reaktoro. Additional results for the membrane area of the RO unit and the operating pressure of the feed pump are provided in the Supporting Information.

**Figure 7:**
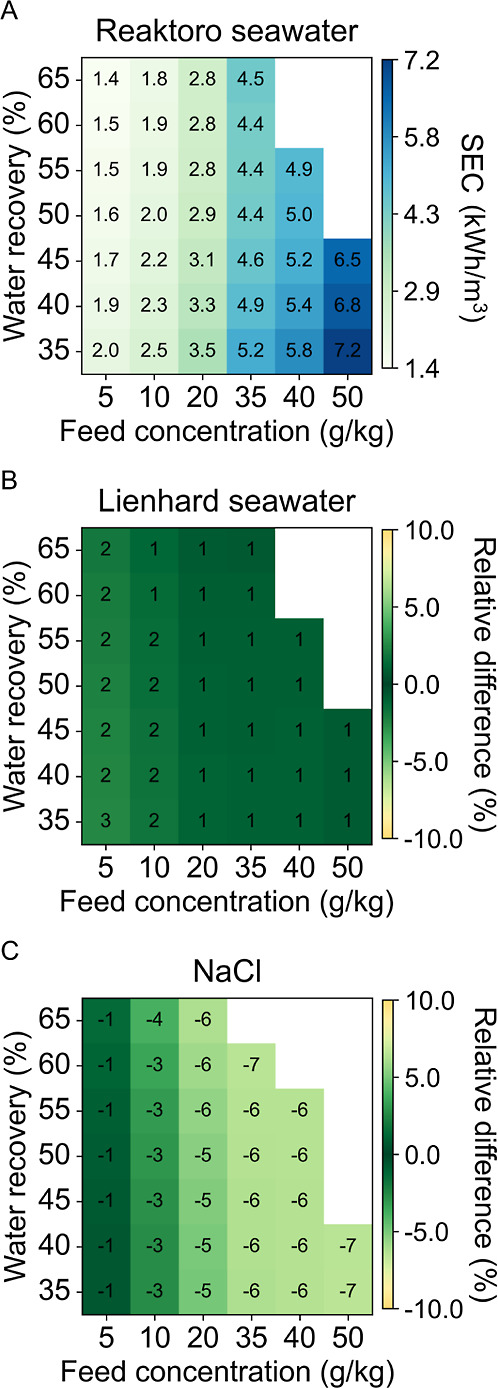
SEC of RO case study for (A) Reaktoro seawater property model and the relative difference of (B) Lienhard seawater property model and (C) sodium chloride property model when compared to Reaktoro.

#### 3.2.2 Mechanical Vapor Compression Cost Optimization Results

The model achieves minimal LCOW by optimizing key MVC decision variables, such as the preheater and evaporator areas, and the compressor pressure ratio. The preheater areas enhance energy efficiency by utilizing waste heat, while the evaporator area influences process capacity. The compressor pressure ratio determines the energy required for vapor compression, directly impacting overall performance. Alongside these decision variables, the evaluation relies on specific outcome metrics that assess both performance and process design. Performance metrics include LCOW and SEC, while process metrics focus on design and operational parameters. Understanding these metrics enables a more detailed analysis of how variations in the property model influence the optimal design. Figures depicting the results for the evaporator area and compressor pressure ratio are available in the Supporting Information.

Figure 8 visually illustrates the LCOW results for the MVC case study, comparing three seawater property models: (A) Reaktoro seawater property model, (B) Lienhard seawater property model, and (C) sodium chloride property model. The Lienhard seawater and sodium chloride models yield similar quantitative results, a maximum 3% increase in LCOW. This close alignment suggests that the fundamental thermodynamic behaviors are accurately characterized across these models. Additionally, the precision in vapor pressure and enthalpy calculations adds to the models’ reliability, establishing a strong theoretical basis for optimization.

**Figure 8:**
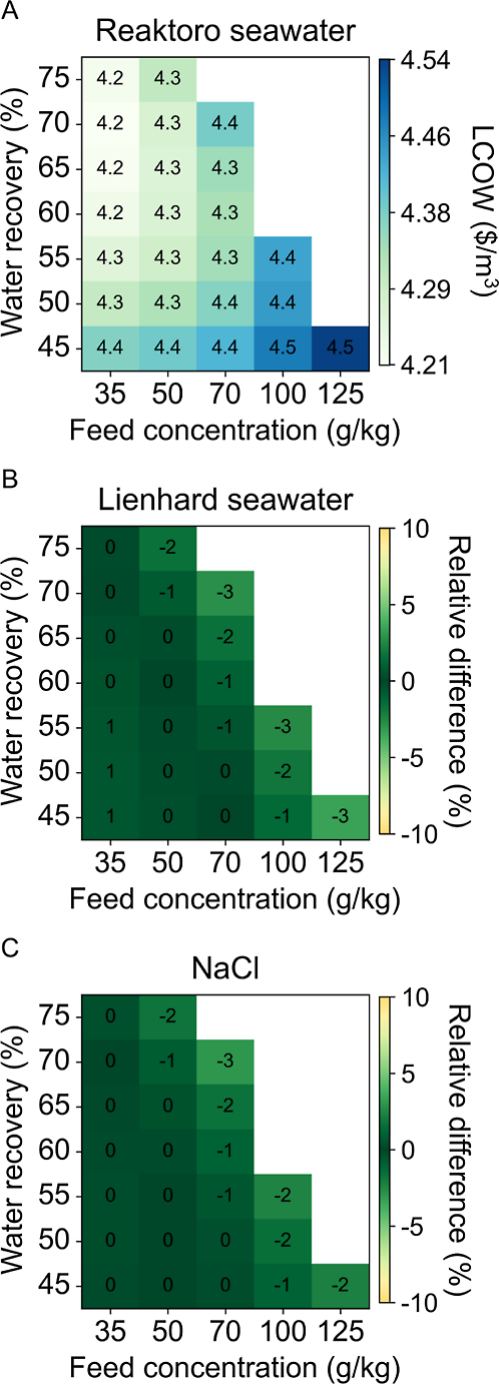
LCOW of MVC case study for (A) Reaktoro seawater property model and the relative difference of (B) Lienhard seawater property model and (C) sodium chloride property model when compared to Reaktoro.

Figure 9 presents the SEC results for the MVC case study across all three considered property models. The Lienhard seawater property model exhibits a maximum relative difference of an 8% increase in SEC, while the sodium chloride property model shows a maximum relative difference of a 6% increase in LCOW. The most significant deviations occur as the brine concentration increases, where the validity range is being exceeded. For these highly concentrated cases, the relative differences among the property models are approximately double in the SEC results compared to the LCOW results.

**Figure 9:**
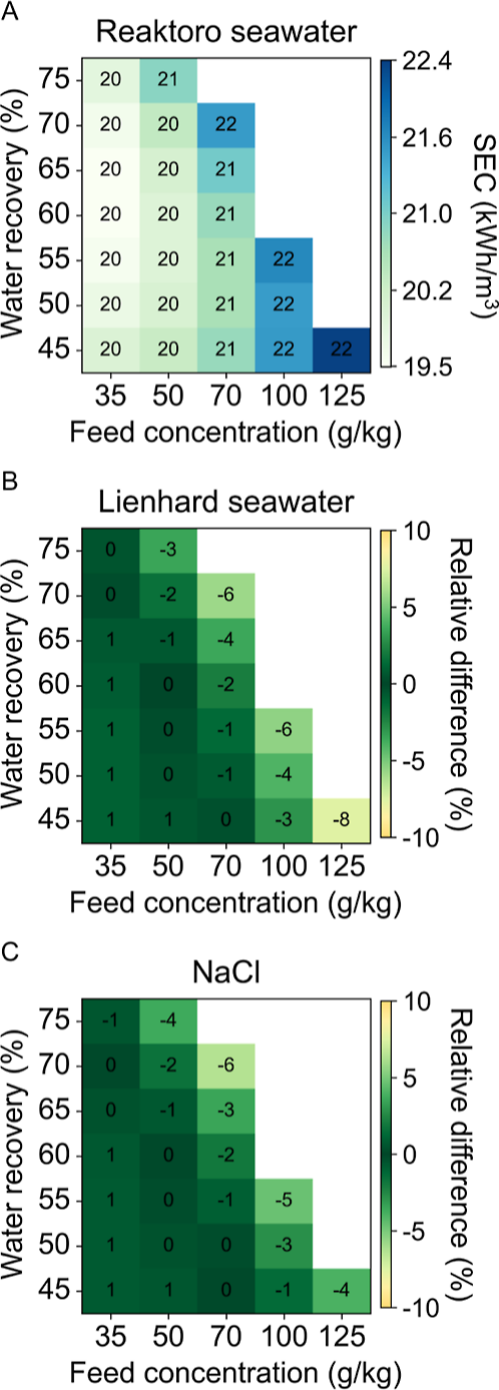
SEC of MVC case study for (A) Reaktoro seawater property model and the relative difference of (B) Lienhard seawater property model and (C) sodium chloride property model when compared to Reaktoro.

### 3.3 Computational Performance

A comparison of computational times for the property models is presented in Table 3. Reaktoro performs detailed thermodynamic calculations specific to the feedwater composition, and Reaktoro-pse configures these equilibrium calculations as gray-box models for seamless integration with WaterTAP, which increases the computational time. In contrast, the Lienhard seawater and sodium chloride models rely on empirical correlations tailored to their respective water types. These methodological differences lead to significant variations in computational time. While the Lienhard seawater and sodium chloride models show similar performance in both RO and MVC case studies, Reaktoro is considerably slower, with execution times 9.6 and 28 times longer, respectively.

**Table 3:** Computational Time Comparison for Property Model Calculations

**RO (41 cases optimized)**	**total run time (s)**
Reaktoro seawater	231
Lienhard seawater	24
Sodium chloride	26

**MVC (31 cases optimized)**	**total run time (s)**
Reaktoro seawater	954
Lienhard seawater	34
Sodium chloride	31

### 3.4 Implications for Process Modeling and Optimization

In process modeling and optimization, choosing a property model involves balancing simplicity with accuracy. Empirical models, such as the Lienhard seawater and sodium chloride models, accurately estimate seawater properties within their specific validity ranges. Their computational efficiency and ease of use make them ideal for tasks requiring quick calculations, such as preliminary design studies and sensitivity analyses. For these common applications, a simpler model is usually the most practical option. However, these simpler models have a significant limitation: they break down when a process operates outside their defined concentration or temperature ranges. Advanced tools, such as Reaktoro/Reaktoro-pse, then become essential. Reaktoro can handle complex, multicomponent chemistry. It performs detailed speciation calculations, predicting how various chemical species interact under a wide range of conditions, making it suitable for modeling complex water compositions, estimating scaling potentials, and performing chemical additions. These models offer a more comprehensive and accurate view of a complex system. Ultimately, no single model is suitable for every situation. Combining the strengths of both empirical and detailed thermodynamic models offers the most robust solution.

## 4 Conclusions

This paper provided a comprehensive comparison of three property models for seawater: the Reaktoro multicomponent seawater model, the Lienhard bulk total dissolved solids seawater model, and the sodium chloride model. All three models yield similar predictions for seawater properties. For typical seawater desalination applications, the Lienhard seawater and sodium chloride models deliver sufficiently accurate results. The Reaktoro model remains valuable for applications where empirical models are not easily accessible, such as softening, scaling predictions, and pH adjustment.

Future work should prioritize an integrated approach that combines the strengths of both empirical and detailed thermodynamic methods, providing a robust and versatile framework for process modeling. Additionally, future research should investigate the incorporation of process operability, which assesses design and control objectives simultaneously,^27^ into the optimization framework. This approach ensures maximum operational capacity and efficiency while optimizing process design and control strategies for improved performance. By incorporating these aspects, future studies can develop models that not only predict seawater properties accurately but also enhance overall process design and control.

## Supplementary Material



## Notes

Additional modeling details, including model equations, costing information, and figures showing additional results for RO and MVC process design variables (PDF)
