# Supplementary material for: Assessing the Accuracy of Property Model Predictions for Cost Optimization of Desalination Technologies
Source: ACS ES T Eng. 2026 Jan 23;6(2):793–801. doi: 10.1021/acsestengg.5c00929 (PMC12910588; doi:10.1021/acsestengg.5c00929)
Supplement: Supplementary file 1 [file ee5c00929_si_001.pdf]

Assessing the accuracy of property model predictions for cost optimization of desalination technologies

Savannah S. Sakhai,† Timothy V. Bartholomew,‡ Alexander V. Dudchenko,‡ and Fernando V. Lima*,†

†*Department of Chemical and Biomedical Engineering, West Virginia University, Morgantown, WV 26506, USA*

‡*National Energy Technology Laboratory, Pittsburgh, PA 15236, USA*

E-mail: Fernando.Lima@mail.wvu.edu

S1 Suplementary Information

S1.1 Additional modeling details

The details for equations and implementation of all unit, property, and cost models used in this work can be found on WaterTAP documentation[1] and Github repository.[2] Additional details for how Reaktoro was integrated with WaterTAP can be found on the reaktoro-pse Github repository.[3]

S1.2 RO case study: additional figures

The RO cost optimization case study in the main text examines the performance metrics, LCOW and SEC. The decision variables of the optimization, namely the membrane area

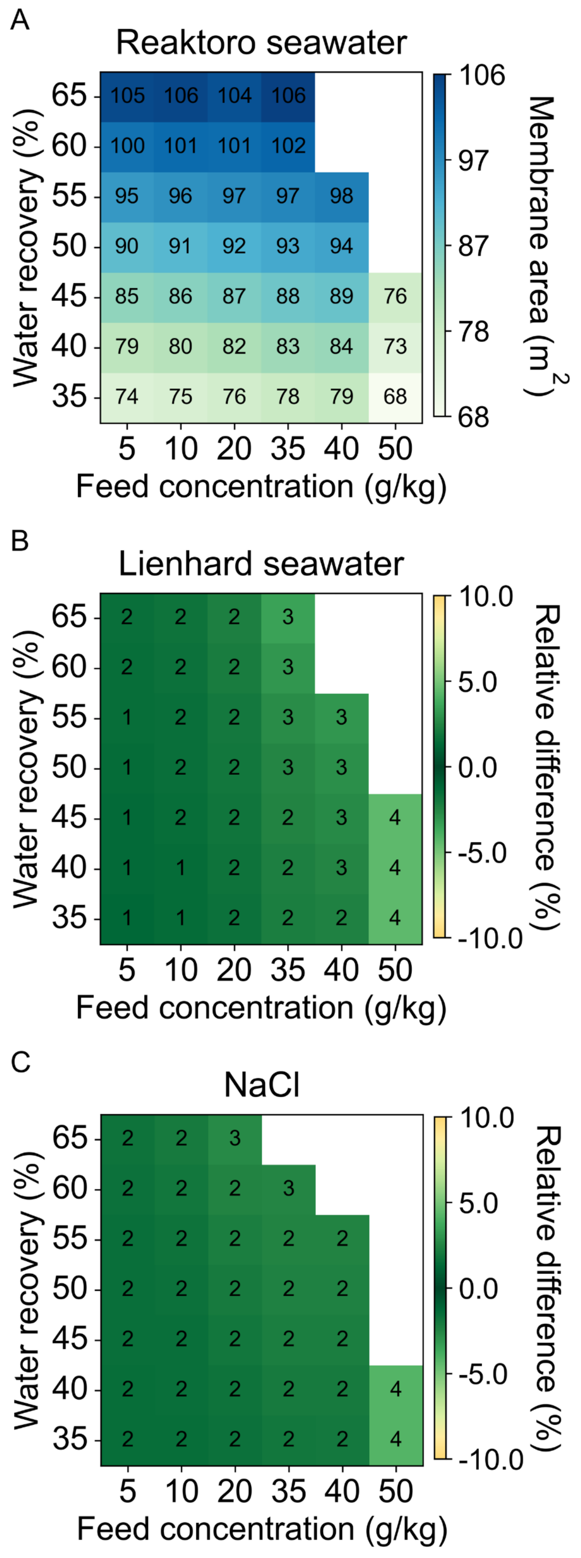

Figure S1. Membrane area of RO case study for A) Reaktoro seawater property model and the relative difference of B) Lienhard seawater property model and C) sodium chloride property model when compared to Reaktoro.

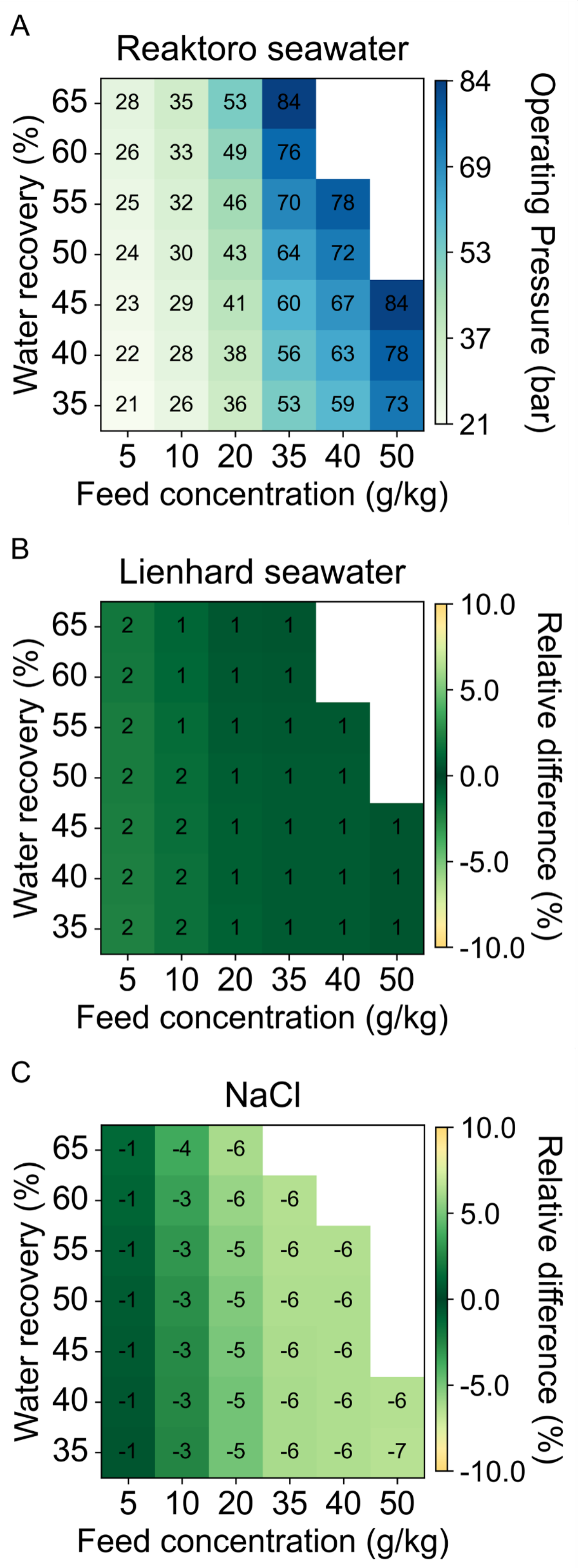

Figure S2. Operating pressure of RO case study for A) Reaktoro seawater property model and the relative difference of B) Lienhard seawater property model and C) sodium chloride property model when compared to Reaktoro.

and operating pressure, illustrate how the system design directly varies according to the optimization process. This enables comparisons to be made across solutions for each property model. Figure S1 depicts the membrane area resulting from cost optimization for three distinct seawater property models: A) the Reaktoro seawater model, B) the Lienhard seawater model, and C) the sodium chloride model. The membrane area increases with higher feed concentration and water recovery, as demonstrated in Figure S1A, which presents the Reaktoro seawater results. When comparing these results with the Lienhard seawater results shown in Figure S1B, there is a slight relative difference ranging from 1% to 4%. The sodium chloride results in Figure S1C exhibit similar trends, with relative differences from 2% to 4%. Figure S2 illustrates the operating pressure resulting from cost optimization across the three aforementioned seawater property models: A) the Reaktoro seawater model, B) the Lienhard seawater model, and C) the sodium chloride model. The operating pressure rises with higher feed concentration and water recovery, as shown in Figure S1A, where the Reaktoro seawater results align with the membrane area trend. In comparison to the Lienhard seawater results in Figure S1B, the relative difference is minimal, ranging from 1% to 2%. The sodium chloride results in Figure S1C display similar outcomes, with relative differences ranging from -7% to -1%.

S1.3 MVC case study: additional figures

The MVC cost optimization case study outlined in the main text assesses performance metrics, notably LCOW and SEC. The decision variables for optimization, such as the evaporator area and compressor pressure ratio, highlight how the optimization process directly influences system design. This facilitates comparisons of solutions for each property model. Figure S3 illustrates the evaporator area achieved through cost optimization for three different seawater property models: A) the Reaktoro seawater model, B) the Lienhard seawater model, and C) the sodium chloride model. The evaporator area increases with greater feed concentration and water recovery, as shown in Figure S3A for the Reaktoro seawater results.

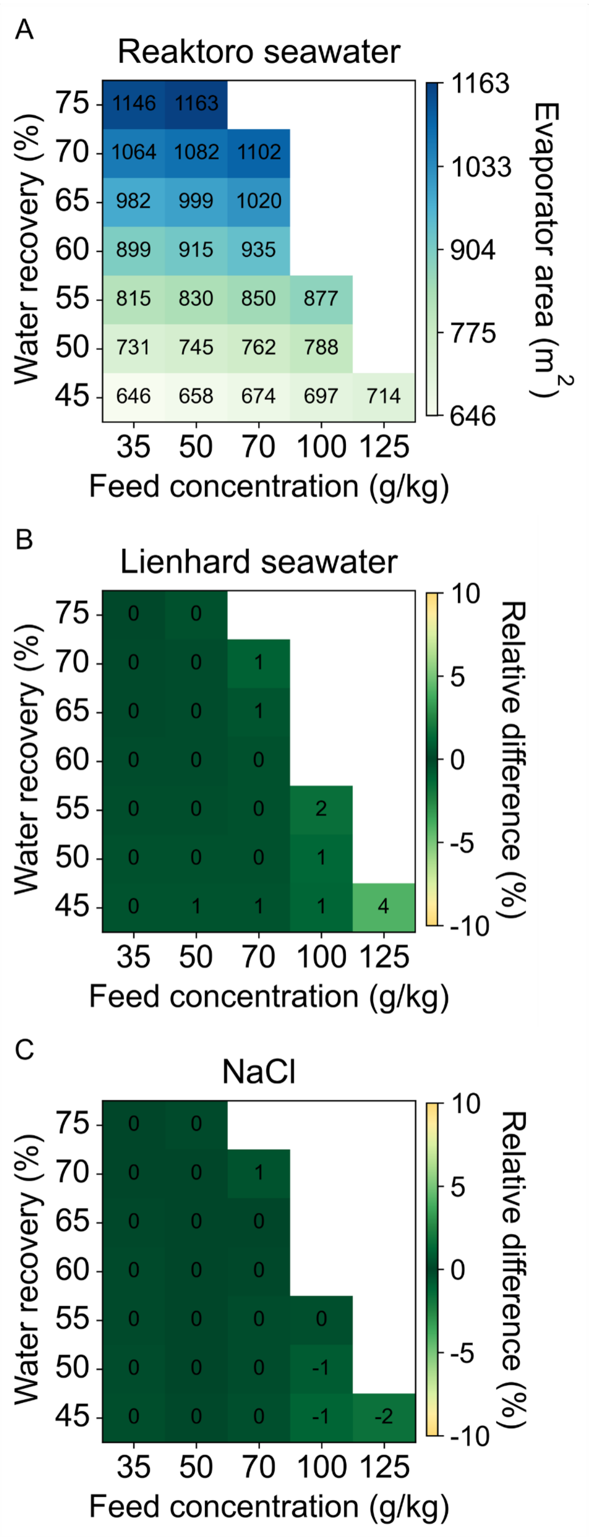

Figure S3. Evaporator area of MVC case study for A) Reaktoro seawater property model and the relative difference of B) Lienhard seawater property model and C) sodium chloride property model when compared to Reaktoro.

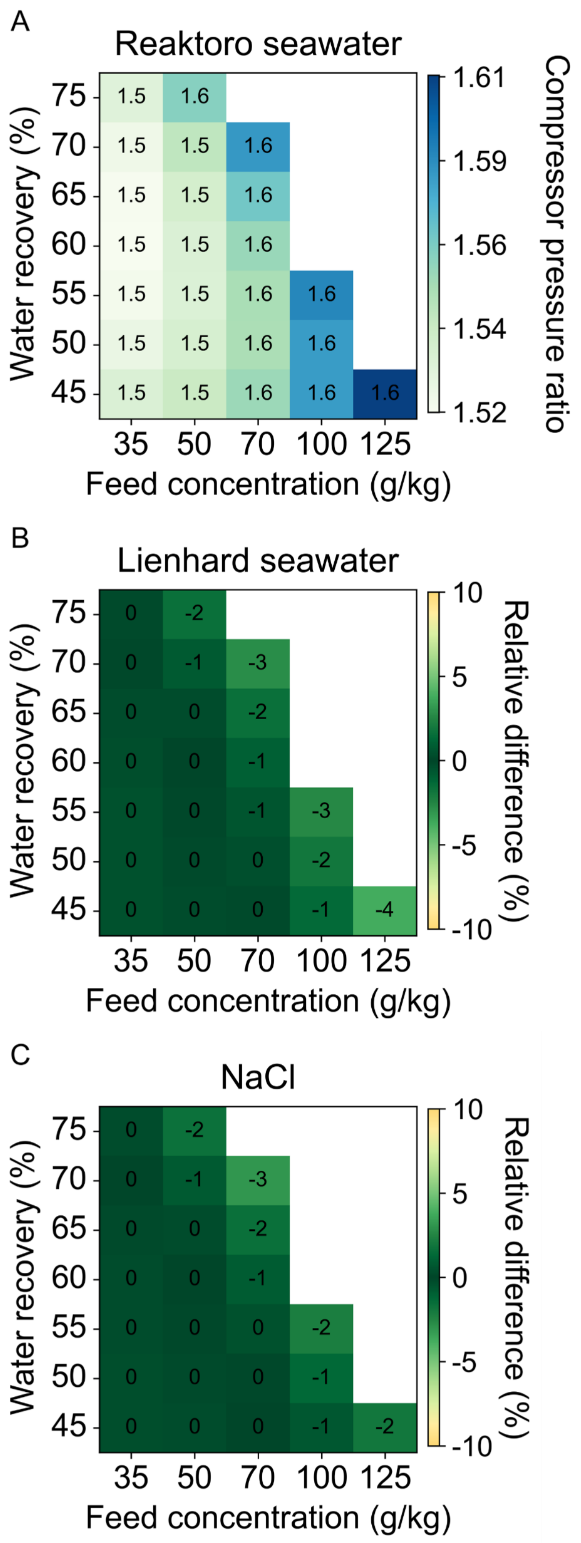

Figure S4. Compressor pressure ratio of MVC case study for A) Reaktoro seawater property model and the relative difference of B) Lienhard seawater property model and C) sodium chloride property model when compared to Reaktoro.

When these results are compared with those of the Lienhard seawater model in Figure S3B, a slight relative difference of 0% to 4% is observed. The sodium chloride results in Figure S3C demonstrate similar trends, with relative differences ranging from -2% to 0%. Figure S4 depicts the compressor pressure ratio from cost optimization across the same three seawater property models: A) the Reaktoro seawater model, B) the Lienhard seawater model, and C) the sodium chloride model. The compressor pressure ratio increases with higher feed concentration and water recovery, reflected in Figure S4A, where the Reaktoro seawater results correspond to the trend seen in the evaporator area. In comparison with the Lienhard seawater results in Figure S4B, the relative difference is minor, ranging from -4% to 0%. The sodium chloride results in Figure S4C yield similar findings, showing relative differences from -3% to 0%.

References

(1) WaterTAP 1.3.0 Documentation. `https://watertap.readthedocs.io/en/stable/`.

(2) Atia, A.; Holly, M.; Knueven, B.; Wang, C.; Amusat, O.; Barber, H.; Beattie, K.; Bi, X.; Bianchi, L.; Binger, Z.; V. Dudchenko, A.; Garciadiego, A.; Gunter, D.; Lee, A.; Panda, K.; Pesce, M.; Sakhai, S.; Shamlou, E.; A-Sitterley, K.; Vecchiarelli, P.; Allu, S.; Hardikar, M.; Mauter, M.; Tucker, C.; Bartholomew, T. *WaterTAP 1.0 Release*; 2024.

(3) Dudchenko, A. V. reaktoro-pse. 2025; `https://github.com/watertap-org/reaktoro-pse`.
